# Supplementary material for: A Label-Free Colorimetric Aptasensor for Flavokavain B Detection
Source: Sensors (Basel). 2025 Jan 19;25(2):569. doi: 10.3390/s25020569 (PMC11768667; doi:10.3390/s25020569)
Supplement: Supplementary file 1 [file sensors-25-00569-s001.zip › sensors-3404015-supplementary.pdf]

A Label-Free Colorimetric Aptasensor for Flavokavain B Detection

Sisi Ke [1,†] Ningrui Wang [2,†] Xingyu Chen [1], Jiangwei Tian [1,*], Jiwei Li [1,*], and Boyang Yu [1]

[1] Jiangsu Key Laboratory of TCM Evaluation and Translational Research, School of Traditional Chinese Pharmacy, China Pharmaceutical University, Nanjing 211198, P.R. China; wy893406895@163.com (S.K.); chenxingyugs@163.com (X.C.); boyangyu59@163.com (B.Y.)

[2] Nanchang Medical College, Nanchang, Jiangxi 330052, China; aurora_0711@163.com (N.W.)

* Correspondence: jwtian@cpu.edu.cn (J.T.); jiweili@cpu.edu.cn (J.L.)

† These authors contributed equally to this work.

Table S1. Detailed DNA sequences employed in this research.

Name	Sequence (5′ to 3′)
DNA library	CGAGCATAGGCAGAACTTACGAC(N30)GTCGTAAGAGCGAGTCATTC
cDNA-bio	TTTTTGTCGTAAGTTCTGCCATTTT/3STGBio/
FP	CGAGCATAGGCAGAACTTAC
RP-bio	/5STGBio/GAATGACTCGCTCTTACGAC
FKB-1	CGAGCATAGGCAGAACTTACCACCAAGTGAGGGTGTACGGTGGCTTACGC TTGGTCGTAAGAGCGAGTCATTC
FKB-2	CGAGCATAGGCAGAACTTACCACGAGGACGGGTCAGTGCGTTAGGTGCCC ACAGTCGTAAGAGCGAGTCATTC
FKB-3	CGAGCATAGGCAGAACTTACCACACAGCGTACCTCGTAGCCATGCGCCGTT GGGTCGTAAGAGCGAGTCATTC
FKB-1-5′FAM	/5SFAM/CGAGCATAGGCAGAACTTACCACCAAGTGAGGGTGTACGGTGGCT TACGCTTGGTCGTAAGAGCGAGTCATTC
FKB-S	CTTACCACCAAGTGAGGGTGTACGGTGGCTTACGCTTGGTCGTAAG
FKB-S-5′FAM	/5SFAM/CTTACCACCAAGTGAGGGTGTACGGTGGCTTACGCTTGGTCGTAAG

Table S2. Detailed information regarding the conditions of SELEX.

Round#	Counter-targets	Counter-target Concentration	FKB (μM)
1-2	-	-	500
2-3	-	-	250
4-5	-	-	125
6-9	myricetin, quercetin, aloe emodin, isoglycyrrhizin	100	125
10	myricetin, quercetin, aloe emodin, isoglycyrrhizin, FKA, FKC	100, 50 (FKA, and FKC)	125
11-12	myricetin, quercetin, aloe emodin, isoglycyrrhizin, FKA, FKC	100	125
13-14	myricetin, quercetin, aloe emodin, isoglycyrrhizin, FKA, FKC, hypericin, puerarin, hesperidin, astilbin, catechin, ophiopogon dihydroisoflavone B	100	125
15-16	myricetin, quercetin, aloe emodin, isoglycyrrhizin, FKA, FKC, hypericin, puerarin, hesperidin, astilbin, catechin, ophiopogon dihydroisoflavone B	100	50
17-18	myricetin, quercetin, aloe emodin, isoglycyrrhizin, FKA, FKC, hypericin, puerarin, hesperidin, astilbin, catechin, ophiopogon dihydroisoflavone B	100	10
19	myricetin, quercetin, aloe emodin, isoglycyrrhizin, FKA, FKC, hypericin, puerarin, hesperidin, astilbin, catechin, ophiopogon dihydroisoflavone B	100	5
20-27	FKA, FKC	100	5

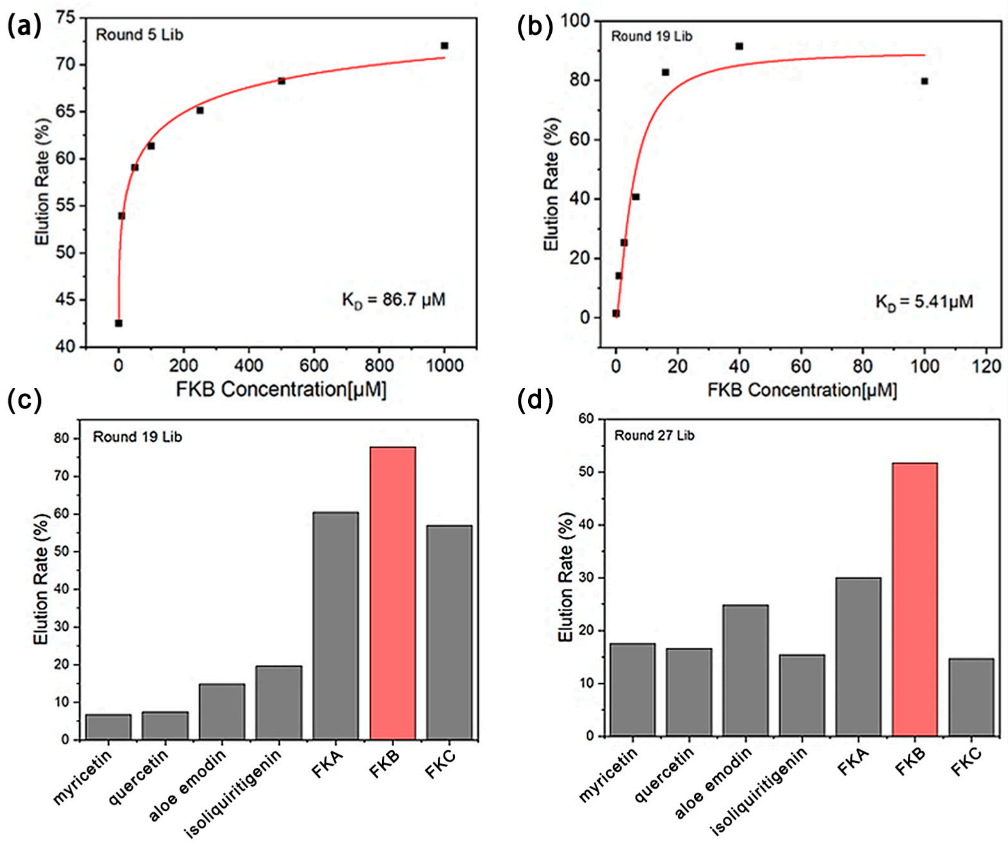

Figure S1. Binding affinity and specificity of 5th pool (a and c) and19th pool (b and d) was determined by Gel elution assay.

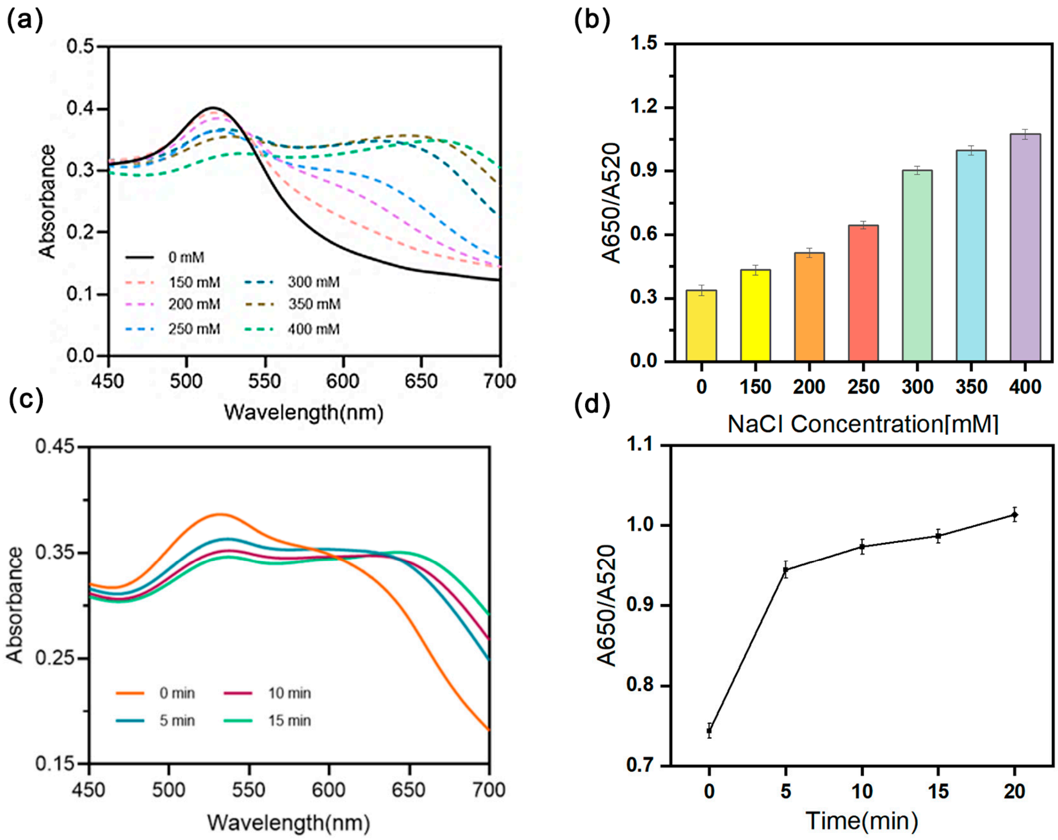

Figure S2. Optimization of NaCl concentration (a and b) and reaction time (c and d) in the aptasensor.
